# Graft failure rate and complications after Descemet membrane endothelial keratoplasty in eyes with pre-existing glaucoma

**DOI:** 10.1007/s00417-022-05813-4

**Published:** 2022-08-30

**Authors:** Anna-Karina B. Maier, Daniel Pilger, Enken Gundlach, Sibylle Winterhalter, Necip Torun, Tina Dietrich-Ntoukas

**Affiliations:** Department of Ophthalmology, Campus Virchow-Klinikum, Charité – Universitätsmedizin Berlin, corporate member of Freie Universität Berlin, Humboldt-Universität zu Berlin and Berlin Institute of Health, Augustenburger Platz 1, 13353 Berlin, Germany

**Keywords:** DMEK, Descemet membrane endothelial keratoplasty, Intraocular pressure elevation, Post-DMEK glaucoma, Post-keratoplasty glaucoma, Graft survival, Graft failure rate

## Abstract

**Purpose:**

To evaluate the outcome of Descemet Membrane Endothelial Keratoplasty (DMEK) in eyes with pre-existing glaucoma.

**Design:**

In this retrospective, observational case series we included data of 150 consecutive DMEKs in eyes with pre-existing glaucoma of 150 patients after excluding data of the second treated eye of each patient and of re-DMEKs during follow-up. Cumulative incidences of IOP elevation (IOP > 21 mmHg or ≥ 10 mmHg increase in IOP from preoperative value), post-DMEK glaucoma (need of an additional intervention due to worsening of the IOP), graft rejection, and graft failure rate were analyzed using Kaplan–Meier survival analysis. COX regression analysis was used to evaluate independent risk factors.

**Results:**

The 36-month cumulative incidence of IOP elevation was 53.5% [95 CI 43.5–63.5%] and of post-DMEK glaucoma 36.3% [95 CI 26.3–46.3%]. Graft rejection occurred with a 36-month cumulative incidence of 9.2% [CI 95% 2.3–16.1]. None of the analyzed risk factors increased the risk for the development of graft rejection. The 36-month cumulative incidence of graft failure was 16.6% [CI 95% 8.4–24.8]. Independent risk factors for graft failure were the indication for DMEK “status after graft failure” (*n* = 16) compared to Fuchs’ dystrophy (*n* = 74) (*p* = 0.045, HR 8.511 [CI 95% 1.054–68.756]) and pre-existing filtrating surgery via glaucoma drainage device (GDD) (*n* = 10) compared to no surgery/iridectomy (*n* = 109) (*p* = 0.014, HR 6.273 [CI 95% 1.456–27.031]).

**Conclusion:**

The risks of postoperative complications (IOP elevation, post-DMEK glaucoma, graft rejection, and graft failure) in patients with pre-existing glaucoma are high. In particular, pre-existing filtrating surgery via GDD implantation—but not trabeculectomy—and DMEK after graft failure increase the risk of graft failure.


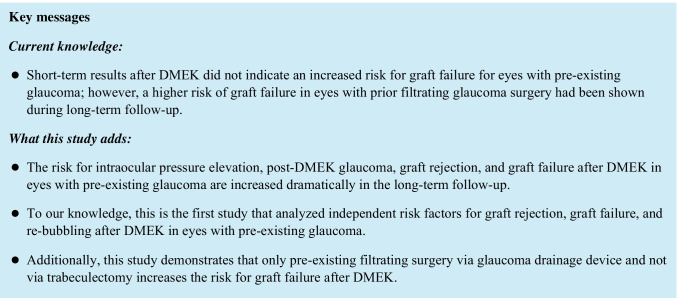


## Introduction

Descemet membrane endothelial keratoplasty (DMEK) has been established as effective treatment for corneal endothelial disorders such as Fuchs’ corneal endothelial dystrophy and bullous keratopathy after cataract surgery. Compared to perforating keratoplasty and Descemet stripping (automated) endothelial keratoplasty (DS(A)EK), DMEK shows excellent clinical outcome, low complication rate, and a rapid visual rehabilitation [1–4]. Due to these advantages, the indication for DMEK expanded in recent years. In more complicated cases such as graft failure after previous keratoplasty and bullous keratopathy after multiple glaucoma surgeries, DMEK surgery is nowadays also performed successfully [5–8].

The important influence of pre-existing glaucoma on corneal graft survival is known for procedures as perforating keratoplasty and DS(A)EK. Reinhard et al. showed that eyes with pre-existing glaucoma had a significant lower 3-year graft survival rate than eyes without glaucoma after perforating keratoplasty (71% versus 89%) [9]. After DS(A)EK, the 4-year graft survival was also significant lower in eyes with pre-existing glaucoma than in those without [10]. Additionally, eyes which were treated with glaucoma medication only had a significantly higher graft survival rate than eyes with filtrating glaucoma surgery prior to DS(A)EK (96% versus 69% after 4 years) [10]. Short-term results after DMEK did not indicate an increased risk for graft failure [8, 11]. However, a higher risk of graft failure in eyes with prior filtrating glaucoma surgery had been shown during long-term follow-up [12–18].

A negative influence of postoperative intraocular pressure (IOP) elevation on the graft endothelium is discussed as one of the main reasons, in addition to effects of anti-glaucomatous medication, mechanical damage caused by glaucoma drainage devices (GDD) or need for additional surgeries [9]. As shown by a previous study, eyes with pre-existing glaucoma had a higher risk for IOP elevation and post-DMEK glaucoma (postoperative secondary glaucoma with the need of an additional intervention due to worsening of the IOP) during long-term follow-up [19].

Therefore, we investigated the long-term postoperative complications including the cumulative incidences of postoperative IOP elevation, post-DMEK glaucoma, graft rejection, and graft failure after DMEK in patients with pre-existing glaucoma over a 36-month time period. Additionally, we analyzed independent risk factors of graft rejection and graft failure.

## Patients and methods

### Patients

In this retrospective, cohort, outcome study between September 2011 and July 2019, we included eyes from patients with pre-existing glaucoma who underwent DMEK surgery at the Department of Ophthalmology, Charité – Universitätsmedizin Berlin consecutively. Data of the second treated eye of each patient and of re-DMEKs during follow-up were excluded. Ethic approval had been given by the Ethikkommission, Charité – Universitätsmedizin Berlin, EA4/167/16. For this retrospective, single-center study formal consent was not required; the Ethikkommission, Charité – Universitätsmedizin Berlin approved the waiver of consent. The study adhered to the ethical standards of the Declaration of Helsinki. An informed written consent was provided for surgery.

Clinical examinations were performed preoperatively and postoperatively after 4 weeks, 3, 6, 12, 24, 36, 48, and 60 months after DMEK. Data after graft failure were not included in the study. The examinations included: best corrected visual acuity (BCVA) tested with a Snellen chart, slit lamp examination, single IOP measurement using Goldmann’s applanation tonometry (Haag Streit, Bern, Switzerland), endothelial cell density (ECD) measured by specular microscope Nidek CEM-530 (NIDEK Co. Ltd. Japan) and fundoscopy. The Snellen decimal number was converted into logMAR visual acuity using a conversion table. Corneal thickness was not considered for IOP measurement according to results of Maier et al. [20]. In addition, in some cases, pneumatic tonometer (CT20D computerized Tonometer, Topcon, Japan) was used for IOP measurement. When the IOP was elevated using pneumatic tonometry an additional measurement with Goldmann’s applanation tonometry was performed. The preoperative IOP measurement with Goldmann’s applanation or pneumatic tonometry was in 10 cases not reliably possible due to a preoperative corneal edema; therefore, the IOP was measured by palpation. Whenever feasible, the cup to disc ratio (C/D) was documented and a peripapillary retinal nerve fibre layer (RNFL) thickness measurement by Spectralis optical coherence tomography (OCT) (Spectralis OCT, Heidelberg Engineering GmbH, Heidelberg, Germany) was performed preoperatively or at the first two visits postoperatively. Additionally, medical history of every patient was reviewed including age, gender, pre-DMEK diagnoses, prior history of glaucoma, and postoperative glaucoma treatment.

Glaucoma diagnosis and classification was based on the criteria of the European Glaucoma Society and definition of pre-existing glaucoma included following criteria: documented history of glaucoma, prior glaucoma filtration surgery, preoperative use of glaucoma medication, and typical glaucomatous excavation of the optic disc or Cup/Disc ratio ≥ 0.6 (categorized by two independent observers). For COX regression analysis, preoperative glaucoma surgery was categorized in four groups: group 1: no previous glaucoma surgery or iridotomy/iridectomy, group 2: IOP lowering filtrating surgery via GDD implantation (Ahmed or Baervelt valve, XEN stent), group 3: IOP lowering filtrating surgery via trabeculectomy, group 4: all other glaucoma surgeries and glaucoma classification was categorized in four groups: group 1: primary open-angle glaucoma/high pressure glaucoma and normal-pressure glaucoma, group 2: primary angle-closure glaucoma, group 3: (pseudo)exfoliation glaucoma, group 4: all others including pigmentary glaucoma, aphakic glaucoma, uveitic glaucoma, other secondary glaucoma or congenital glaucoma.

IOP elevation after DMEK was defined as described by Maier et al. [21] as IOP ≥ 22 mmHg or an increase in IOP from preoperative value ≥ 10 mmHg at any postoperative examination and all eyes with postoperative elevated IOP were categorized respectively [21]. In short, eyes with steroid-induced glaucoma were defined as eyes in which the IOP normalized (≤ 21 mmHg) when the steroid treatment was ceased, post-DMEK glaucoma as postoperative secondary glaucoma with the need of an additional intervention due to worsening of the IOP. Rare cases with IOP elevation due to postoperative pupillary block (< 1%) were not included in this study.

Occurrence of keratitic precipitates with corneal edema with or without cells in the anterior chamber or with or without ciliary injection defined graft rejection. An irreversible corneal cloudiness or edema (concomitant with insufficient corneal transparency for adequate vision) defined graft failure. The time at which the patient presented with the findings at our clinic was defined as the time of graft rejection or graft failure. An early graft failure was defined as a persisting edema after surgery despite additional air injections (re-bubbling) within 12 weeks after surgery.

### Graft and surgical technique

Previous studies described in detail the used graft and surgical technique [19] and the standard postoperative topical treatment. Organ cultured grafts from the Cornea Bank Berlin, Charité – Universitätsmedizin Berlin with a minimum central endothelial density of 2000/mm^2^ were transplanted. The diameter of the graft was 7.5 – 9.0 mm.

Elevated postoperative IOP was treated as described by Maier et al. [19, 21].

If indicated, re-bubbling (injection of air in the anterior chamber) was performed in the early postoperative period for graft detachments of more than 2 clock hours with corneal edema in more than 2 clock hours.

### Treatment of postoperative IOP elevation

First treatment option for all patients with postoperative IOP elevation was a medical therapy (mainly eye drops) to control the IOP.

In steroid-induced glaucoma, normalizing of IOP was achieved by tapering down topical steroids and giving additional glaucoma medication to treat IOP elevation.

Post-DMEK glaucoma was treated by additional topical therapy or an additional surgical intervention.

### Statistical methods

Statistical analysis was performed using IBM SPSS statistics 19 (SPSS Software, Munich, Germany). Normality was tested for all outcome measures and the appropriate statistical test was used. Descriptive statistics were expressed as median and range or mean ± standard deviation (SD). We used Kaplan–Meier survival analysis to estimate cumulative incidences. Graft rejection was defined as 1—graft rejection-free survival and graft failure as 1—graft survival. Data of patients after their last presentation in our clinic are censored. In order to identify independent risk factors, we built a COX regression based on bivariable associations with graft rejection or graft failure. All possible risk factors were checked for an association with the outcome and were included in the COX regression if we found evidence for an association (*p* value < 0.05). Possible risk factors included preoperative RNFL, preoperative IOP, pre-existing glaucoma according to the mentioned glaucoma classification, pre-existing glaucoma surgery, preoperative glaucoma medication with carbonic anhydrase inhibitors, preoperative diagnosis as indication for DMEK, post-DMEK glaucoma, postoperative tapering steroid eye drops < 1 year, re-bubbling, or graft rejection. For patients with need for re-bubbling, we checked the risk factors pre-existing glaucoma according to the glaucoma classification, pre-existing glaucoma surgery, preoperative diagnosis as indication for DMEK, type of DMEK surgery (DMEK alone or combined) and patients and donor age for an association. The proportional hazard assumption was checked and a *p* value of < 0.05 was considered statistically significant.

## Results

We reviewed 201 eyes of 150 patients. Data of 51 eyes were excluded because of the exclusion criteria “second eye” and “re-DMEK” which were performed within the study period. Finally, 150 eyes of 150 patients (mean age 71.7 ± 13.3 years) were included in the study. Mean follow-up was 26.1 ± 22.4 months. 75.3% reached a minimum 340 days follow-up and 28.0% a minimum 1050 days follow-up without graft failure.

All surgeries were performed by three experienced surgeons (NT, TDN, AKM), who completed the learning curve of minimum 50 DMEK surgeries beforehand. We summarized the demographic, surgical, and preoperative results in Table 1. Preoperative IOP was ≥ 22 mmHg in 8 of 140 patients (5.7%). In the remaining 10 of 150 patients, preoperative IOP measurement could only be measured by palpation and measurement was normotensive.

**Table 1:** Demographic, surgical, and preoperative results

	Total (*n* = 150)
Age (years, mean ± SD)	71.7 ± 13.3 (*n* = 150)
Indication for DMEK	
% Fuchs’ corneal dystrophy	49.3% (*n* = 74)
% bullous keratopathy	40.0% (*n* = 60)
% graft failure	
After perforating keratoplasty	6.0% (*n* = 9)
After DSAEK	4.0% (*n* = 6)
After DMEK	0.7% (*n* = 1)
Surgery	
DMEK	75.3% (*n* = 113)
Triple-DMEK (phacoemulsification + IOL-implantation + DMEK)	20.0% (*n* = 30)
DMEK with IOL change	4.7% (*n* = 7)
Preoperative VA (LogMAR, mean ± SD)	1.09 ± 0.59 (*n* = 150)
Endothelial cell density (cells/mm^2^, mean ± SD)	2268 ± 193 (*n* = 147)
Pre-existing glaucoma surgery	
No	66.7% (*n* = 100)
Iridotomy/Iridectomy	6.0% (*n* = 9)
IOP lowering surgery:	
Filtrating surgery/trabeculectomy	14.0% (*n* = 21)
Filtrating surgery/glaucoma drainage device (Ahmed or Baervelt valve, XEN stent)	6.7% (*n* = 10)
Others (trabectome, istent inject, canaloplasty, Cypass, cyclodestructive procedures)	6.7% (*n* = 10)
Glaucoma classification	
Group 1	
Primary open-angle glaucoma / High pressure glaucoma	54.7% (*n* = 82)
Primary open-angle glaucoma / Normal-pressure glaucoma	2.7% (*n* = 4)
Group 2	
Primary angle-closure glaucoma	6.7% (*n* = 10)
Group 3	
Pseudoexfoliative glaucoma	14.7% (*n* = 22)
Group 3	
Pigmentary glaucoma	2.0% (*n* = 3)
Uveitic glaucoma	6.7% (*n* = 10)
Aphacic glaucoma	2.7% (*n* = 4)
Other secondary glaucoma (open-angle glaucoma due to intraocular tumor, due to ocular trauma, due to ocular surgery) or congenital glaucoma	10.0% (*n* = 15)
Number of glaucoma medications	
0	8.7% (*n* = 13)
1	42.0% (*n* = 63)
2	27.3% (*n* = 41)
3	18.0% (*n* = 27)
4	4.0% (*n* = 6)
Preoperative IOP (mmHg, mean ± SD)	15.1 ± 4.2 (*n* = 140)
Preoperative RNFL (µm, mean ± SD)	82.2 ± 18.3 (*n* = 112)
Cup-disc ratio (mean ± SD)	0.7 ± 0.2 (*n* = 120)

### Postoperative results

#### Visual acuity and endothelial cell density

Mean visual acuity improved significantly after surgery (*p* < 0.001, preoperative 1.09 ± 0.59 (*n* = 150) LogMAR versus 0.46 ± 0.53 LogMAR after 12 months (*n* = 112) and 0.39 ± 0.44 LogMAR after 36 months (*n* = 38).

Mean endothelial cell density decreased from 2268/mm^2^ ± 193/mm^2^ (*n* = 147 grafts) to 1739 ± 573/mm^2^ after 12 months (*n* = 44) and 1551 ± 393/mm^2^ after 36 months (*n* = 14).

#### IOP elevation and post-DMEK glaucoma

The 12-month and 36-month cumulative incidences of post-surgical IOP elevation and post-DMEK glaucoma are shown in Table 2.

**Table 2:** Cumulative incidence of postoperative intraocular pressure changes calculated by Kaplan–Meier analysis

	12-month cumulative incidence [95% confidence interval (CI)]	36-month cumulative incidence [95% confidence interval (CI)]
IOP elevation after DMEK (study criteria ≥ 22 mmHg or ≥ 10 mmHg from preoperative IOP)		
IOP elevation (except cases with IOP elevation due to postoperative pupillary block)	39.2% [95 CI 31.0–47.4%]	53.5% [95 CI 43.5–63.5%]
Steroid-induced glaucoma	21.0% [95 CI 13.7–28.3%]	27.4% [95 CI 18.2–36.6%]
Post-DMEK glaucoma	26.3% [95 CI 18.9–33.7%]	36.3% [95 CI 26.3–46.3%]

#### Treatment of postoperative IOP elevation

In 12 of the 30 eyes (40%) of steroid-induced glaucoma, the IOP rose again after topical steroids had been tapered down and these eyes developed a post-DMEK glaucoma as defined before [21, 22].

In total, 43 eyes developed a post-DMEK glaucoma, 27 eyes without pre-existing glaucoma surgery or iridotomy/iridectomy, one eye with GDD implant, 12 eyes after preoperative trabeculectomy and 3 eyes after other glaucoma surgery. Topical therapy was sufficient to control the IOP in 19 eyes (44%) with post-DMEK glaucoma. In 24 eyes (56%) with post-DMEK glaucoma, an additional surgical intervention was necessary to control the IOP. Performed surgical interventions included in 10 eyes filtrating surgeries (trabeculectomy or XEN stent implantation), in 7 eyes cyclodestructive interventions (cyclophotocoagulation), in one eye trabectome procedure and in another eye iStent inject® implantation. In 5 eyes, multiple surgical interventions were performed including filtrating surgery, cyclodestructive procedures and interventions at the trabecular meshwork.

#### Re-bubbling

In 34 eyes (22.7%), a re-bubbling procedure was performed. In 29 of these eyes, one re-bubbling procedure was necessary to achieve a graft re-attachment, in 3 eyes two, in one eye three, and in another eye four re-bubbling procedures. After re-attachment, the corneal edema decreased in 29 eyes during the following 2 to 3 weeks. In 5 other cases, the corneal edema persisted. In two of the cases, intraoperative unfolding and attachment of the flap were not possible due to very flat anterior chamber combined with extensive vis à tergo during the surgery. In these eyes a perforating keratoplasty was performed during the follow-up period. Three other eyes showed no reduction of the corneal edema despite attached flap and received a re-DMEK procedure.

None of the analyzed risk factors, pre-existing glaucoma according to the glaucoma classification (*p* = 0.332, *χ*^2^ = 3.411, df 3), pre-existing glaucoma surgery (*p* = 0.735, *χ*^2^ = 1.276, df 3), preoperative diagnosis as indication for DMEK (*p* = 0.319, *χ*^2^ = 2.285, df 2), type of DMEK surgery (alone or combined) (*p* = 0.762, χ^2^ = 0.544, df 2) and patients (*p* = 0.982) and donor age (*p* = 0.079), showed a significant association with re-bubbling.

#### Graft rejection

**Fig. 1:**
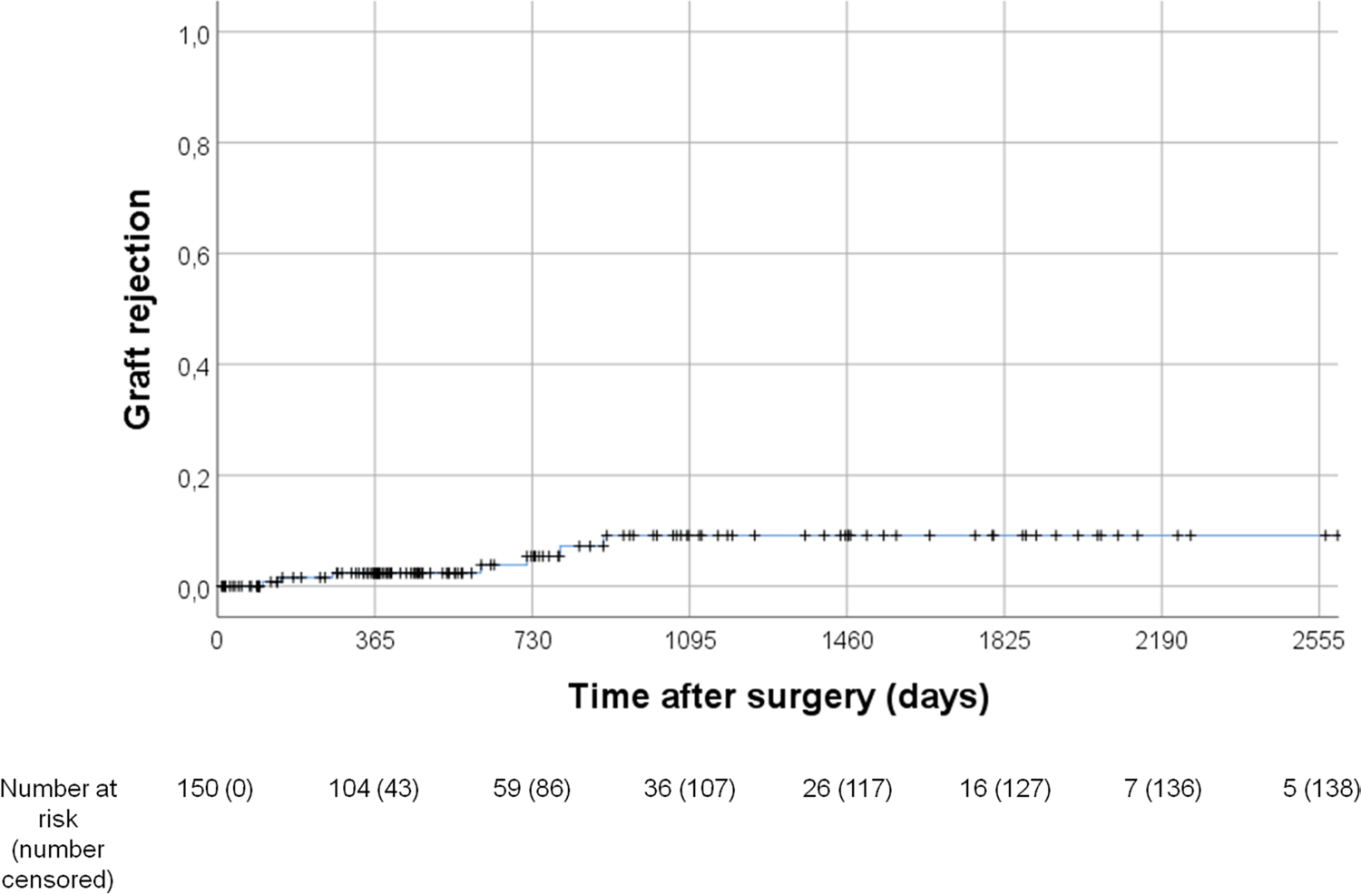
Kaplan–Meier curve of graft rejection (1—graft rejection-free survival) for all eyes

Graft rejection occurred with a 12-month cumulative incidence of 2.4% [CI 95% -0.3−5.1] and a 36-month cumulative incidence of 9.2% [CI 95% 2.3–16.1]. Kaplan–Meier curve of graft rejection is shown in Fig. 1. None of the analyzed risk factors increased the risk for the development of a graft rejection, Table 3.

**Table 3:** Bivariable associations with immune graft rejection or graft failure

	Graft rejection (2 categories: no/yes)	Graft failure (2 categories: no/yes)
Preoperative RNFL (µm)	*p* = 0.819	*p* = 0.280
Preoperative IOP (mmHg)	*p* = 0.110	*p* = 0.102
Glaucoma classification (4 Groups 1 POAG, 2 PACG, 3 pseudoexfoliative glaucoma, 4 all others)	*p* = 0.726 (*χ*^2^ = 1.312, df 3)	***p* < 0.001** (*χ*^2^ = 37.749, df 3)
Pre-existing glaucoma surgery (4 groups: 1 no or iridotomy/iridectomy, 2 filtrating surgery via GDD implantation, 3 filtrating surgery/trabeculectomy, 4 all others)	*p* = 0.770 (*χ*^2^ = 1.131, df 3)	***p* = 0.001** (*χ*^2^ = 16.156, df 3)
Preoperative antiglaucomatous medication (2 categories: carbonic anhydrase inhibitors no/yes)	*p* = 0.584 (*χ*^2^ = 0.300, df 1)	***p* = 0.027** (*χ*^2^ = 4.875, df 1)
Indication for DMEK (3 categories: Fuchs’ corneal dystrophy, bullous keratopathy, graft failure)	*p* = 0.810 (*χ*^2^ = 0.421, df 2)	***p* = 0.004** (*χ*^2^ = 11.004, df 2)
Post-DMEK glaucoma (2 categories: no/yes)	*p* = 0.088 (*χ*^2^ = 2.912, df 1)	***p* = 0.005** (*χ*^2^ = 7.826, df 1)
Re-bubbling (2 categories: no/yes)	*p* = 0.588 (*χ*^2^ = 0.294, df 1)	***p* = 0.010** (*χ*^2^ = 6.566, df 1)
Steroids tapering down within first year (2 categories: no/yes)	*p* = 0.827 (*χ*^2^ = 0.048, df 1)	*p* = 0.866 (*χ*^2^ = 0.352, df 1)
Graft rejection (2 categories: no/yes)	-	*p* = 0.939 (*χ*^2^ = 0.006, df 1)

Bold letters indicate *p *< 0.05

*RNFL*, retinal nerve fiber layer; *IOP*, intraocular pressure; *POAG*, primary open-angle glaucoma; *PACG*, primary angle-closure glaucoma; *DMEK*, Descemet membrane endothelial keratoplasty; *GDD*, glaucoma drainage device

#### Graft failure

**Fig. 2:**
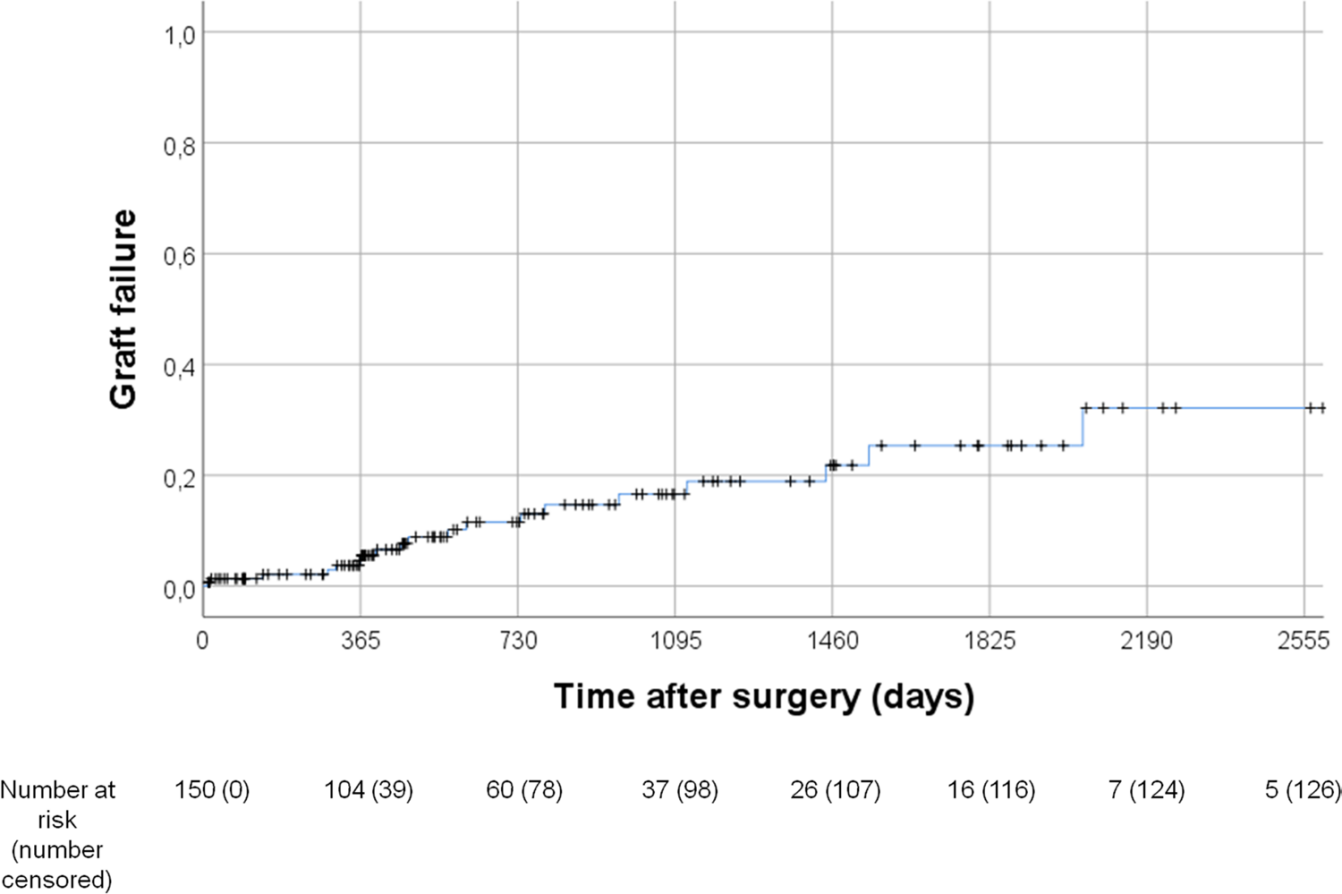
Kaplan–Meier curve of graft failure (1—graft survival) for all eyes

The 12-month cumulative incidence of graft failure was 5.5% [CI 95% 1.4–9.6], the 36-month cumulative incidence 16.6% [CI 95% 8.4–24.8]. Kaplan–Meier curve of graft failure is shown in Fig. 2. Five eyes developed an early graft failure concomitant with persisting edema after surgery as described for the re-bubbling procedure. In all other eyes, graft failure occurred later than 12 weeks after surgery. Bivariable associated risk factors for graft failure are presented in Table 3. Independent risk factors for graft failure were the indication for DMEK graft failure compared to FED patients (*p* = 0.045, HR 8.511 [CI 95% 1.054–68.756]) and the pre-existing glaucoma surgery filtrating surgery via GDD implantation compared to no surgery/iridectomy (*p* = 0.014, HR 6.273 [CI 95% 1.456–27.031]) in the COX regression analysis. Preoperative glaucoma medication, glaucoma diagnosis, re-bubbling, and post-DMEK glaucoma did not influence the risk for graft failure (*p* > 0.05).

## Discussion

After DMEK surgery, in eyes with pre-existing glaucoma, IOP elevation occurs in more than 50 percent of patients and a third of patients develops a post-DMEK glaucoma. In these eyes, 3-year incidence of graft rejection is approx. 9%, but not increased by any analyzed risk factor. Graft failure occurs with a 3-year incidence of approximately 17%. A pre-existing filtrating surgery via GDD implantation, but not trabeculectomy, and the indication for DMEK “status after graft failure” increased the risk of graft failure. However, the re-bubbling rate was not increased and influenced by any risk factor, not even by the kind of pre-existing glaucoma surgery.

### IOP elevation and post-DMEK glaucoma

Twelve-month incidence of post-surgical IOP elevation ranges from 6 to 16% and of post-DMEK glaucoma from 2 to 4% in patients after DMEK surgery [19, 21–27]. In patients with pre-existing glaucoma, the risk for both is significantly increased as previously shown: 12-month cumulative incidence of IOP elevation 39.2% [95 CI 31.0–47.4%] and of post-DMEK glaucoma 26.3% [95 CI 18.9–33.7%] [19]. After 36 months, the cumulative incidences for both still increased and reached 53.5% [95 CI 43.5–63.5%] for IOP elevation and 36.3% [95 CI 26.3–46.3%] for post-DMEK glaucoma. Several other studies showed also that pre-existing glaucoma is the main risk factor for post-surgical IOP elevation and development of a post-DMEK glaucoma [9, 10, 19, 22–27].

In this study, steroid-induced IOP elevation was a common cause for postoperative IOP elevation, but proportionally less often than in other DMEK patients [19, 22, 28]. Additionally, we found that in 40% of eyes with steroid-induced IOP elevation, the IOP rose again after tapering down topical steroids and these eyes developed a post-DMEK glaucoma. Therefore, other reasons such as e.g. exacerbation of pre-existing glaucoma may play also a role in these patients.

Topical therapy was sufficient to control the IOP in only 44% of eyes with post-DMEK glaucoma, whereas 56% needed an additional surgical intervention. The need of a surgical treatment of post-DMEK glaucoma in eyes with pre-existing glaucoma as described previously [19, 22] is probably due to the fact that patients with pre-existing glaucoma require a lower target pressure. Additionally, there are often fewer treatment options due to the fact that patients are already under topical therapy.

### Graft rejection and graft failure

Graft rejection rate after DMEK is increased for patients with pre-existing glaucoma with a 3-year incidence of approx. 9% compared to published data with a graft rejection rate between 2.3 and 2.6% after 4–7 years [2, 29, 30]. Different studies demonstrated also high graft rejection rates between 17.2 and 20.8% in eyes that had previously undergone trabeculectomy and/or GDD implantation after 4 years [13–15, 18].

Reasons for the high graft rejection rate may be that steroids have to be reduced earlier due to a high incidence of steroid-induced IOP elevation as shown by our data. Additionally, postoperative IOP elevation itself, which occurred very often in patients with pre-existing glaucoma, may trigger graft rejection as shown for perforating keratoplasty. Analyzing our data, we found no independent risk factor for graft rejection. Neither the earlier tapering down of the postoperative steroids nor a postoperative IOP elevation nor pre-existing factors increased the risk of a graft rejection. Both Boutin et al. and Sorkin et al. discussed a more aggressive postoperative steroid regime because of a high rejection rate and a low steroid response rate in eyes that had previously undergone trabeculectomy and/or GDD implantation [13, 14] while in both studies, systematic analysis of postoperative steroid-induced IOP elevation is missing. Compared to these data, our study demonstrated a lower graft rejection rate and a high 3-year incidence of steroid-induced IOP elevation (27.4%). Therefore, a general recommendation for an aggressive postoperative steroid regime of patients with pre-existing glaucoma should be critically assessed and further studies are required [28].

For perforating keratoplasty and DS(A)EK as well as DMEK, graft failure rate in patients with pre-existing glaucoma is increased [9, 10, 19, 31]. Our data are in accordance with these findings with a 3-year incidence of approximately 17%. Compared to data in the literature with a graft failure rate between 3.1 and 3.6% 7–8 years after DMEK [2, 30], this represents a significant elevation. Data of Sorkin et al., Bonnet et al., and Alshaker et al. also showed high graft failure rates in eyes that had previously undergone trabeculectomy and/or GDD implantation [14, 15, 18]. These publications showed an increased graft failure rate in eyes with previous filtrating glaucoma surgery compared to eyes without and with only medically treated eyes [15]. To analyze this aspect and other possible reasons for an increased graft failure rate in eyes with pre-existing glaucoma, we used the COX regression analysis. In contrast to the previous presented results, filtrating surgery (trabeculectomy and/or GDD implantation) was primarily no independent risk factor. When analyzing both groups separately, filtrating surgery via GDD implantation increased the risk of graft failure significantly while filtrating surgery via trabeculectomy does not. Regarding pre-existing glaucoma surgery, the ratio in our filtrating surgery group was shifted in favor of trabeculectomy (2/3 of eyes) compared to glaucoma drainage devices (1/3 eyes) while previous studies showed the opposite ratio [13–15]. Therefore, both filtrating surgeries should be judged differently in terms of their effect on the endothelial cell loss and graft survival. It may not be the filtrating effect that is decisive, but the mechanical effect of the GDD, which induces dysfunction of the endothelial cells. In eyes without graft failure, however, the endothelial cell loss after 36 months was 33.9%, which is in the normal range compared to other studies [30, 32]. Other studies showed significant differences in endothelial cell loss between eyes with a previous glaucoma surgery and a control group only in the long-term follow-up [8, 14, 16], while especially GDD are well known for high endothelial loss rates [33]. As discussed by Sorkin et al., failed grafts cannot be included in endothelial cell density analysis, therefore the endothelial cell loss in our study is probably underestimated [14]. Additionally, the total number of eyes with GDD is low in our study collective (*n* = 10).

The preoperative indication for DMEK “status after graft failure” was the second significant independent risk factor for graft failure. As shown by other studies, these patients have a higher risk for an additional graft failure [34].

The preoperative glaucoma medication with carbonic anhydrase inhibitors, glaucoma classification, preoperative IOP, post-DMEK glaucoma, or re-bubbling did not influence the risk for graft failure. The impact of re-bubbling on the endothelial cell density and graft failure rate had been analyzed before and different studies showed an increased endothelial cell loss after re-bubbling but no reduced graft survival [12, 35–37] as shown by our data. The re-bubbling rate itself (22.7%) was not higher compared to studies of centers using the same re-bubbling strategy [19]. None of the analyzed risk factors, especially the pre-existing glaucoma surgery, showed a significant association with re-bubbling. This is in accordance to other studies which showed no higher re-bubbling rates compared to a control group in eyes that had previously undergone trabeculectomy and/or GDD implantation [13–15, 38]. The preoperative IOP was not correlated to the graft failure rate in our study, although this association is well-known [19]. Probably this is due to the fact that we had a low rate of patients with bad regulated IOP (≥ 22 mmHg) before DMEK surgery.

### Study limitation

The retrospective nature of the study and the wide range of follow-up duration limit the results. Especially, the endothelial cell density can only be assessed to a limited extent because of the fact that a reliable endothelial cell count is not possible in most cases of failed grafts and because of missing data due to missing follow-up examination. As common for a “real world” clinical setting, the cohort is heterogenous concerning pre-operative glaucoma diagnosis, pre- and postoperative glaucoma treatment, and DMEK indication. We took this inhomogeneity into account by using the COX regression analysis. Additionally, there are limiting factors regarding IOP elevation and its measurement, especially in secondary glaucoma after DMEK, which have to be taken into account as discussed in detail before by our group [9, 19–22].

### Conclusions

Pre-existing glaucoma increases the risk of graft rejection and graft survival after DMEK. In particular, a pre-existing filtrating surgery via GDD implantation but not trabeculectomy and a DMEK after graft failure amplify this effect on the risk of graft failure. In conclusion, DMEK surgery can improve vision in most cases of eyes with pre-existing glaucoma, but is associated with a higher complication rate and glaucoma monitoring is mandatory.
